# Virtual Quasi-2D Intermediates as Building Blocks for Plausible Structural Models of Amyloid Fibrils from Proteins with Complex Topologies: A Case Study of Insulin

**DOI:** 10.1021/acs.langmuir.2c00699

**Published:** 2022-05-26

**Authors:** Wojciech Puławski, Wojciech Dzwolak

**Affiliations:** † Institute of High Pressure Physics, Polish Academy of Sciences, 29/37 Sokołowska Str., 01-142 Warsaw, Poland; ‡ Faculty of Chemistry, Biological and Chemical Research Centre, University of Warsaw, 1 Pasteur Str., 02-093 Warsaw, Poland

## Abstract

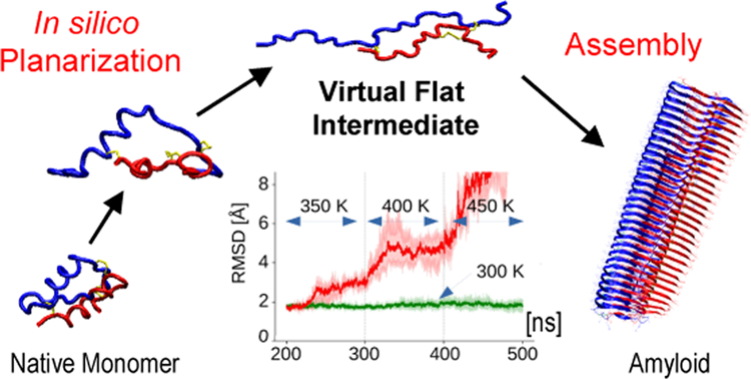

Conformational transitions of globular proteins into amyloid fibrils are complex multistage processes exceedingly challenging to simulate using molecular dynamics (MD). Slow monomer diffusion rates and rugged free energy landscapes disfavor swift self-assembly of orderly amyloid architectures within timescales accessible to all-atom MD. Here, we conduct a multiscale MD study of the amyloidogenic self-assembly of insulin: a small protein with a complex topology defined by two polypeptide chains interlinked by three disulfide bonds. To avoid kinetic traps, unconventional preplanarized insulin conformations are used as amyloid building blocks. These starting conformers generated through uniaxial compression of the native monomer in various spatial directions represent 6 distinct (out of 16 conceivable) two-dimensional (2D) topological classes varying in N-/C-terminal segments of insulin’s A- and B-chains being placed inside or outside of the central loop constituted by the middle sections of both chains and Cys7A–Cys7B/Cys19B–Cys20A disulfide bonds. Simulations of the fibrillar self-assembly are initiated through a biased in-register alignment of two, three, or four layers of flat conformers belonging to a single topological class. The various starting topologies are conserved throughout the self-assembly process resulting in polymorphic amyloid fibrils varying in structural features such as helical twist, presence of cavities, and overall stability. Some of the protofilament structures obtained in this work are highly compatible with the earlier biophysical studies on insulin amyloid and high-resolution studies on insulin-derived amyloidogenic peptide models postulating the presence of steric zippers. Our approach provides *in silico* means to study amyloidogenic tendencies and viable amyloid architectures of larger disulfide-constrained proteins with complex topologies.

## 1 Introduction

Formation of amyloid fibrils, the highly ordered β-sheet-rich aggregates, is a generic structural transition accessible to various proteins and polypeptides.^1−3^ This phenomenon is of paramount biomedical importance. Historically, abnormal amyloid deposits in various tissues have been linked to a wide spectrum of degenerative maladies including Alzheimer’s and Parkinson’s diseases, cardiac amyloidosis, and type II diabetes mellitus.^3−5^ Later studies have shown that early aggregates rather than mature amyloid fibrils may be involved in the etiology of some of these conditions (e.g., refs (6, 7)). On the other hand, there are many recognized examples of biologically functional amyloid fibrils.^8−10^ Formation of amyloid aggregates is often thermodynamically favored under close-to-physiological conditions.^11^ However, *de novo* amyloidogenesis encompasses several discrete stages that may be associated with significant barriers of both enthalpic and entropic nature (for example, partial unfolding of the native state, local association of aggregation-prone intermediates, and formation of amyloid nuclei).^1,3^ The downhill thermodynamics of amyloidogenesis becomes accessible only to proteins acquiring certain levels of instability and at, or above, certain critical concentrations.^12,13^ The last two decades have witnessed significant progress in understanding the molecular and physicochemical mechanisms of amyloidogenesis and its role in biology. While the mainly experimental efforts (including detailed structural studies on amyloid fibrils from typically moderate in length polypeptide chains^14−17^) have contributed to the present state of the field, for a long time, these were paralleled and complimented by various computational approaches (e.g., refs (18−24)). Challenges faced by *in silico* studies on amyloid formation are multiple and significant (see refs (25−28)). Simplistic amyloid models based on the applications of coarse-grained methods lack realistic structural detail and do not address protein–protein and protein–solvent interactions to a satisfying degree. On the other hand, the sheer complexity of the simulated systems exacerbated by high numbers of polypeptide chains (monomers) that need to be considered for physically realistic simulations requires enormous computational resources in the case of all-atom molecular dynamics. This holds true even for implicit solvent simulations that are unlikely to adequately address solvation phenomena known to play an important role in the aggregation process. Therefore, there is a strong argument for undertaking even more costly explicit solvent simulations to probe conformational dynamics of amyloidogenic and transiently hydrated states.^29^ Furthermore, the force fields often used to simulate formation of fibrils may be suboptimally parametrized for this purpose. Especially if a simulation were to cover all of the stages of aggregation of an initially folded and subsequently unfolded (and highly hydrated) protein. The problem of unrealistically high concentrations of proteins subjected to *in silico* aggregation is recognized, as well.^26^ Unsurprisingly, the majority of computational amyloid studies are focused on short peptides lacking complex topological features (e.g., linear Aβ fragments^19,30^). On the other hand, many proteins with complex topologies defined by the presence of multiple disulfide bonds such as insulin, lysozyme, or α-lactalbumin are also amyloidogenic^31−35^ and there is evidence that the native disulfide bonding is preserved in the fibrillar form (e.g., ref (36)). Although there are fewer developed ideas as to how these complex backbone topologies adopt the amyloidal β-sheet motive, one could argue that the presence of these multiple constraints must reduce substantially the accessible conformational space and this, in principle, should make the computational simulations easier. The initial goal of this study was to assess various plausible structures of amyloid fibrils of a model disulfide-constrained amyloidogenic protein: insulin. Our work focuses on a subset of amyloidogenic structures consisting of layers of planarized insulin monomers stacked in an in-register parallel manner. This approach is based on the following symmetry argument: For aggregates of disulfide-constrained proteins with complex topologies, saturation of intermolecular contacts may be achieved by stacking of planarized monomers. However, the low symmetry of such building blocks may require an in-register alignment (and a quasi-translational assembly mode) to facilitate saturation of van der Waals interactions and hydrogen bonds between flattened monomers forming an interchain parallel β-sheet structure. As planarization of a protein molecule with a complex disulfide-constrained structure produces discrete pseudo-two-dimensional (2D) topologies, we compare the stability and properties of various types of insulin amyloid fibrils assembled upon in-register stacking of these distinct 2D topologies.

## 2 Methods

There are three main stages of the multiscale approach to the modeling of insulin amyloid fibril proposed in this study. In the first part, flat (quasi-2D) insulin conformers were simulated using an implicit solvent method. Next, these structures were docked to each other to form layered amyloid-like structures. Finally, all of the resulting amyloid-like structures were validated during long all-atom MD simulations. Substantial parts of modeling (points 1, 2, 3) were carried out using the CHARMM program^37^ and the united-atom representation of protein chains with the Gaussian solvent-exclusion model, EEF1.^38^ All parameters, unless stated otherwise, had default values consistent with the parametrization of the force field (switching function for electrostatic and van der Waals terms between 7.0 and 9.0 Å, distance-dependent dielectric screening). The solvent frictional forces were included by performing the Langevin dynamic with a 5.0 fs^–1^ friction coefficient.

### 2.1 Planarization Protocol

The initial coordinates of an insulin monomer conformer were taken from the hexamer structure of bovine insulin, PDB entry 2A3G.^39^ The conformer was placed over a flat surface composed of 625 attractive dummy beads with fixed positions on a rectangular 125 × 125 Å^2^ grid. As the initial orientation of the insulin monomer toward the surface is likely to affect the course of planarization, we used six distinct starting spatial orientations obtained by stepwise 90° rotations of the monomer around the *X*, *Y*, and *Z* axes (see Figure S1 in the Supporting Information). The dummy beads of the surface were modeled with the attractive part of Lennard–Jones potential with *r*_min_ = 5.0 Å and ε modified every 10 ns (ε = −0.5, −1.0, −3.0, −6.0, −9.0 kcal/mol) in a stepwise manner. The simulation’s temperature was set to 400 K causing unfolding and, eventually, flattening of the monomer. We have carried out 100 such simulations for each initial spatial orientation of the insulin monomer (Figure S1 in the Supporting Information). Finally, 102 properly flattened conformers were selected and clustered into 6 topological groups depending on the placement of the N/C-termini of both A- and B-chains inside or outside of the central topological loop. Subsequently, each flat conformer was subjected to an optimization procedure: minimization (50 steps of steepest descent, SD, method and 50 steps of adopted basis Newton–Raphson, ABNR) and 10 ns long molecular dynamics between two parallel (15 Å apart) surfaces in a manner similar to the flattening process except that only the repulsive part of Lennard–Jones potential was applied. In this way, the conformers became physically relaxed, while their planarity was maintained. Finally, within each topological category, a single conformation was selected based on the Procheck score of Ramachandran dihedral angles compatible with the extended structure.^40^

### 2.2 Protofilament Models

The flattened and energetically optimized insulin conformers were then used as building blocks for the molecular docking process. The first layer (flat conformer of insulin) was placed at the origin, while its identical copy was placed 10 Å apart along the direction perpendicular to the monomer plane. During short dynamics (100 ps), the second docking fragment was pulled toward the first one by applying harmonic distance constraints between corresponding C_α_ carbon atoms of both layers, as in a regular, in-register β-sheet structure. Constraints were removed when adjacent C_α_ carbons were less than 5 Å apart. The secondary structure of individual fragments was constrained during this process by holding the dihedrals near their initial values. The initial layer (docked to) had all C_α_ coordinates fixed, whereas the docking layer had its overall shape constrained (rigid docking). Repeating this procedure with other monomers allows us to build the fibril of the desired length.

### 2.3 All-Atom MD

Molecular dynamics simulations were performed using the AMBER CUDA version and FF15IPQ force field.^41,42^ All initial structures were neutralized with Na^+^/Cl^–^ ions at pH 2 and solvated with SPC/E water molecules in a rectangular box with the smallest distance between any protein atom and the box’s boundary of 16 Å. The size of the generated systems due to solvation varied between 30,053 and 53,024 atoms (9754–16406 water molecules, respectively). There were several stages of the initial equilibration process. First, each system was optimized with 200 steps of the steepest descent algorithm followed by 800 steps of the conjugate gradient method and then heated gradually to 300 K during the first ns with backbone atoms constrained to their starting positions in the NPT ensemble followed by 5 ns long equilibration with only C_α_ atoms constrained. All further production runs were carried out without any constraints imposed on heavy atoms. All bond lengths with hydrogen atoms were constrained with the SHAKE algorithm,^43^ which allows for integration with a 2 fs step. We employed the GPU code of *pmemd* with a real-space cutoff of 8.0 Å and the Langevin dynamics with a collision frequency of 2 ps^–1^. For each FC_1_...FC_6_ topology, three independent 200 ns long simulation runs were conducted. The most conserved structure (i.e. with the lowest RMSD) was subsequently used as a starting conformation for high temperature unfolding simulations and 2 μs long simulations at 300 K.

### 2.4 Implicit Solvent MD of Long Protofilaments

The docking procedure was applied to all of the considered pseudo-2D topologies (FC_1_...FC_6_) to construct protofilaments consisting of 30 layers. For adequate solvation of such long structures in very large periodic boxes filled with water molecules, the computation costs are prohibitive. Hence, we chose to simulate the long fibrils using an implicit solvation approach (EEF1,^38^ details described above). All structures were optimized with 500 steps of the SD minimization algorithm followed by 500 steps of the ABNR method. The MD simulations were carried out for 100 ns, with three independent runs for every topology. The last 10 ns of the simulation were used to acquire structural parameters of long protofilaments.

### 2.5 Definitions

The β-sheet content was defined as a fraction of residues involved in this structure as detected by the Stride algorithm.^44^ RMSD was measured only for C_α_ atoms between the interchain disulfide bridges: residues 7–20 of the A-chain and residues 7–19 of the B-chain. We used the previously described approach to determine the twist of fibrils^45^ in which the angle of rotation is measured as a dihedral angle between two vectors of two consecutive layers. Here, we use sulfur atoms in residues Cys7A and Cys20A and residues Cys7B and Cys19B as two pairs of anchor points. Width and height were measured as average parameters of the bounding box around the monomer. As many fragments of our protofilaments are highly mobile, we chose to conduct the measurements in two regimes: (i) with all of the atoms of the single layer taken into account and (ii) measurements limited to the atoms within the central topological loop. In either case, conformers were aligned to the principal axes, so that the orientation would not influence the results. The average number of water molecules per layer was calculated in two stages. First, we identified atoms forming eventual cavities within all final structures with CAVER software.^46,47^ In the next stage, we searched for and counted water molecules that were within 2.5 Å distance from atoms forming the cavity’s wall. This procedure is accurate only for small clusters of interior water molecules; thus, each case was also visually inspected. The FC_4_ aggregate contains a large cluster of water molecules; hence, in this case, the estimation was based on direct counting.

## 3 Results and Discussion

**Figure 1:**
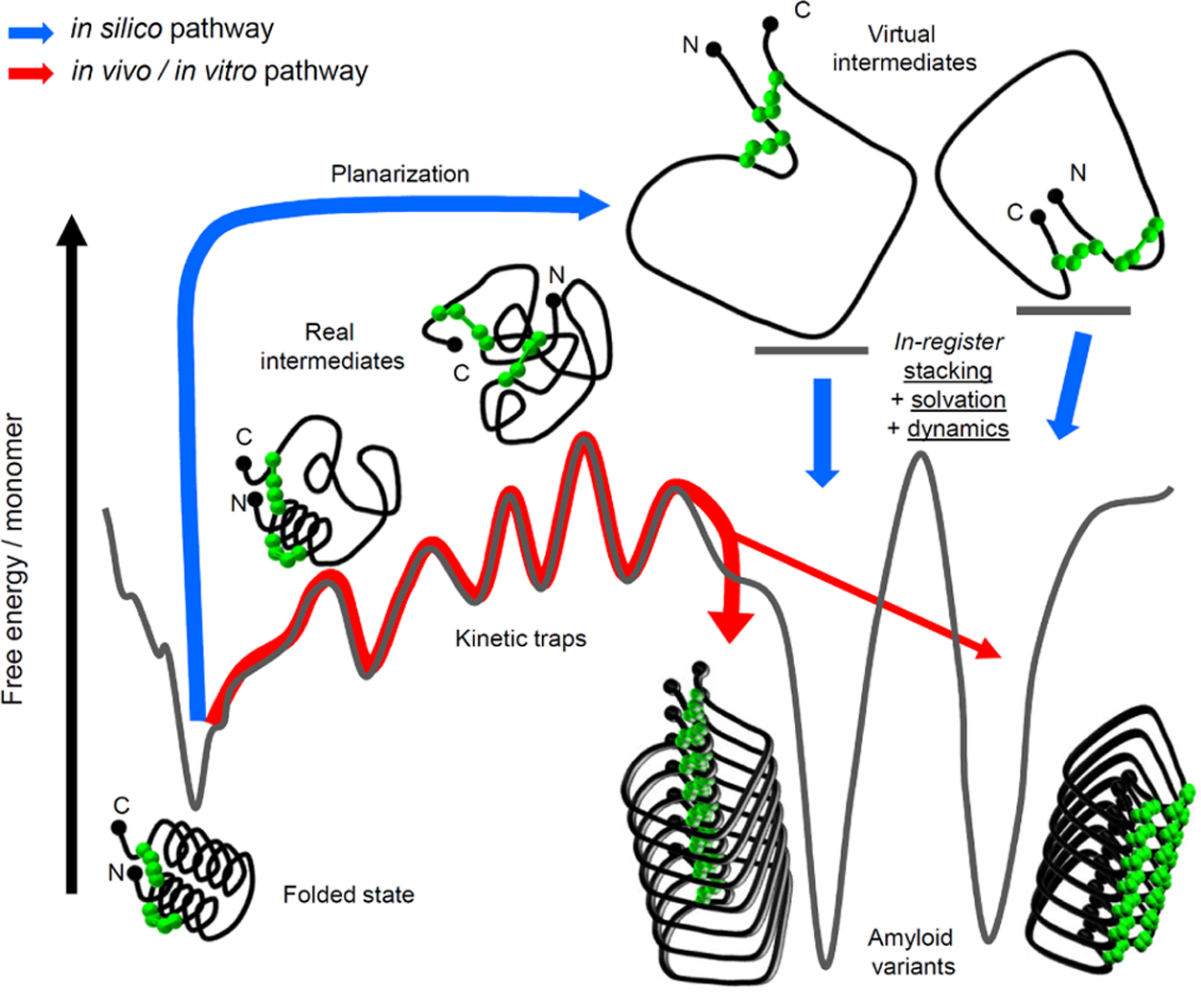
Relationship between the physical and the *in silico* conformational transition pathways leading from a folded protein to amyloid fibrils. The actual transition occurring via partly folded intermediate states is prone to kinetic traps (red pathway). The proposed *in silico* pathway involves initial planarization of the protein molecule to produce highly frustrated virtual intermediates that are subsequently stacked in register to form amyloid fibrils (blue pathway). Two amyloid structural variants distinct in terms of quasi-2D topology of building blocks are considered.

The typical structural and thermodynamic features of amyloid fibrils such as high β-sheet content, high stability, and suppressed backbone fluctuations require that attractive intermolecular interactions within a fibril are effectively saturated. For a macromolecular system consisting of low-symmetry building blocks, one intuitive strategy to achieve this goal in a way that addresses both the short distance nature of van der Waals forces and the directionality of hydrogen bonds is to align individual structural units in a parallel in-register fashion. Following this symmetry argument, we have considered an *in silico* pathway in which planarized protein monomers are initially stacked in this manner. Structures of thus-obtained oligomers are subsequently optimized using molecular dynamics. In Figure 1 depicting the basic idea of this approach, the *in silico* pathway leading from native protein to amyloid fibrils is juxtaposed to the one representing the “real” physical process taking place either *in vivo* or *in vitro*.

While an actual conformational transition of a native protein toward amyloid fibrils is likely to progress through a series of transient partly unfolded intermediates that form the layered structure only at the final stages of aggregation, the *in silico* pathway proceeds through a virtual planarized (a pseudo-2D object) intermediate. Certainly, such an intermediate would not form as a separate entity in solution due to prohibitive energetic and entropic barriers. The *in silico* pathway leading through quasi-2D intermediates mitigates the problems of kinetic traps related to residual native structures and strong local interactions within actual intermediate states. The initial in-register stacking of planar conformations is a biased process of negligible computational cost (in contrast to actual diffusion-limited aggregation of real intermediates in an explicit solvent). The hypothetical protein shown in Figure 1 as a model contains two disulfide bonds. As a result, the flattened virtual intermediates would fall into one of four quasi-2D topological categories depending on whether N- and C-termini are placed inside or outside of the central loop determined by the backbone and the disulfide bonds (for the sake of clarity, only two of these intermediates are shown in Figure 1). Initial assemblies of in-register stacked identical quasi-2D intermediates subsequently undergo structural refinement and optimization through MD. The bovine insulin monomer extracted from the X-ray diffraction structure of the native hexamer (PDB entry: 2A3G(39)) was employed as the starting conformation for the planarization process carried out using CHARMM and implicit solvent environment.^37,38^ The monomer was placed over a flat surface formed by dummy beads fixed on a rectangular grid. The planarization was induced by strong stepwise attraction between the surface and the protein molecule via modified Lennard–Jones potential (see the Methods section). The procedure was carried out 100 times for each of six different initial spatial orientations of the insulin monomer, which could bias the outcome of the planarization process. Indeed, we found that the initial orientation impacts the success rate of the planarization (Figure S1, Supporting Information). We also note that certain initial orientations of the monomer appear to favor particular outcomes of the planarization procedure (i.e., quasi-2D conformations).

**Figure 2:**
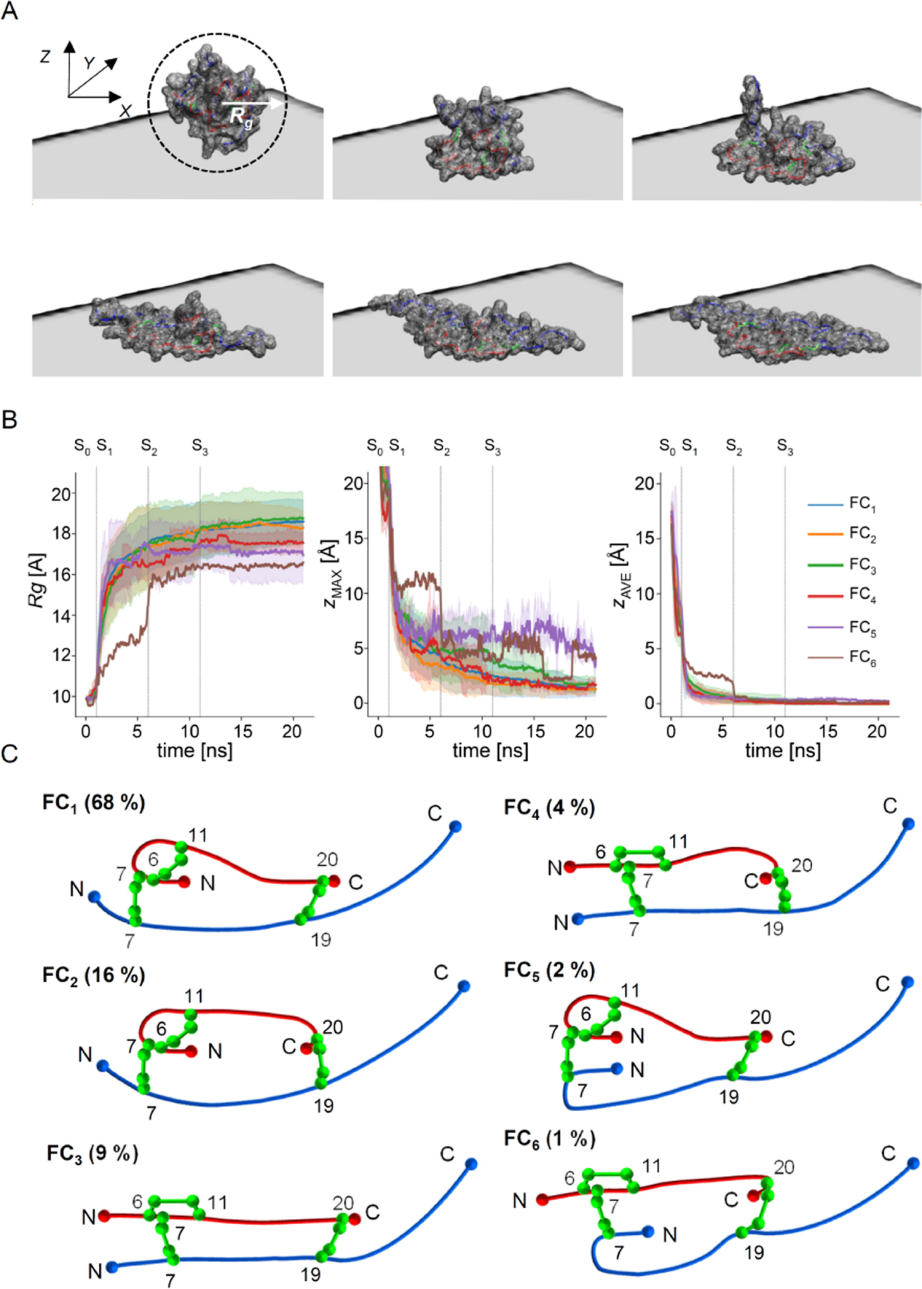
(A) Snapshots of an insulin monomer undergoing the *in silico* planarization process. The initial conformation is the native insulin (PDB: 2A3G); A- and B-chains are colored red and blue, respectively; disulfide bonds are represented as green sticks, and the molecular surface is shown as the gray wireframe (order: from left to right, from top to bottom). (B) Planarization process of the insulin monomer monitored by changes in the radius of gyration (*R*_g_), *Z* coordinate of the center of mass of insulin and maximal value of the protein’s *Z* coordinate. Consecutive steps (*S*_0_, *S*_1_, *S*_2_, *S*_3_) of increasing attraction are marked with the vertical lines, ε = 1, 3, 6, and 9 kcal/mol. (C) Six pseudo-2D topologies of insulin monomer backbones obtained through the *in silico* planarization procedure.

Snapshots of the insulin monomer undergoing the *in silico* planarization are shown in Figure 2A. The molecule unfolds rapidly before becoming completely planar. The progress of the planarization is depicted in Figure 2B by plotting the monomer’s radius of gyration (*R*_g_), the *Z* coordinate of its center of mass, and the thickness (along the *Z* axis) as functions of time. The discrete points of stepwise increases in the surface–monomer attraction are marked as *S*_0_, *S*_1_, *S*_2_, and *S*_3_ (corresponding to −1, −3, −6, and −9 kcal/mol energy). The abrupt increase in *R*_g_ correlates with flattening of the monomer. A successful planarization process would result in a flat conformation in which no segments of the main chains or disulfide bonds overlap each other, i.e., the thickness of the thus-obtained layer should be minimal and uniform and therefore compatible with in-register packing in a quasi-translational pattern. Results of planarization runs that did not meet these criteria were discarded. The filtered results of multiple planarization runs produced 102 flat structures that were clustered into 6 quasi-2D topological groups (marked as FC_1_...FC_6,_ with the decreasing abundance of conformers belonging to given group) distinct in terms of the placement of A- and B-chains’ N- and C-ends relative to the central topological loop of the insulin monomer restricted by the midsections of the main chains and the three disulfide bonds, see Figure 2C. For a hypothetical molecule with zero van der Waals volume and the same as insulin’s type of covalent structure, 16 distinct 2D topologies of the flatten conformers could be envisaged. Among the six observed in our simulations, the most abundant are those in which the N-terminus of A-chain is placed inside the central topological loop: FC_1_ (68%), FC_2_ (16%), FC_5_ (2% of all cases), suggesting that with A-chain’ N-terminal segment pointing inward flat conformations become more accessible. We noticed that a significant portion of the discarded conformers (which were refractory to full planarization) had persistent “bumps” around the Cys7A–Cys7B and Cys6A–Cys11A disulfide bonds and those correlated with A-chain’s N-termini pointing outward. Overall, approximately 86% of all flattened conformers have A-chain’s N-terminal part placed inside the loop: significantly more than the total counts for A-chain’s C-terminal part (21%—see FC_6_, FC_4_, FC_2_) and B-chain’s N-terminal segment (3%—see FC_5_, FC_6_). In none of these simulations, the largest free terminal segment, B-chain’s C-termini, entered the loop of a planarized insulin conformer. It must be stressed that this outcome does not, in itself, prove that such a flat conformer is physically implausible. However, preliminary attempts to force the B-chain’s C-terminal segment into the loop resulted in strained conformations with the dihedral Φ and Ψ angles clearly departing from the canonical values consistent with the β-sheet structure (data not shown). The trajectories reflecting time-dependent changes in monomer’s *R*_g_ and thickness upon uniaxial compression shown in Figure 2B are quite similar regardless of the type of quasi-2D topology to which they ultimately lead.

The distinct appearances of FC_6_ and, to lesser degrees, FC_5_ and FC_4_ cases are most likely due to the low statistics and poor averaging.

**Figure 3:**
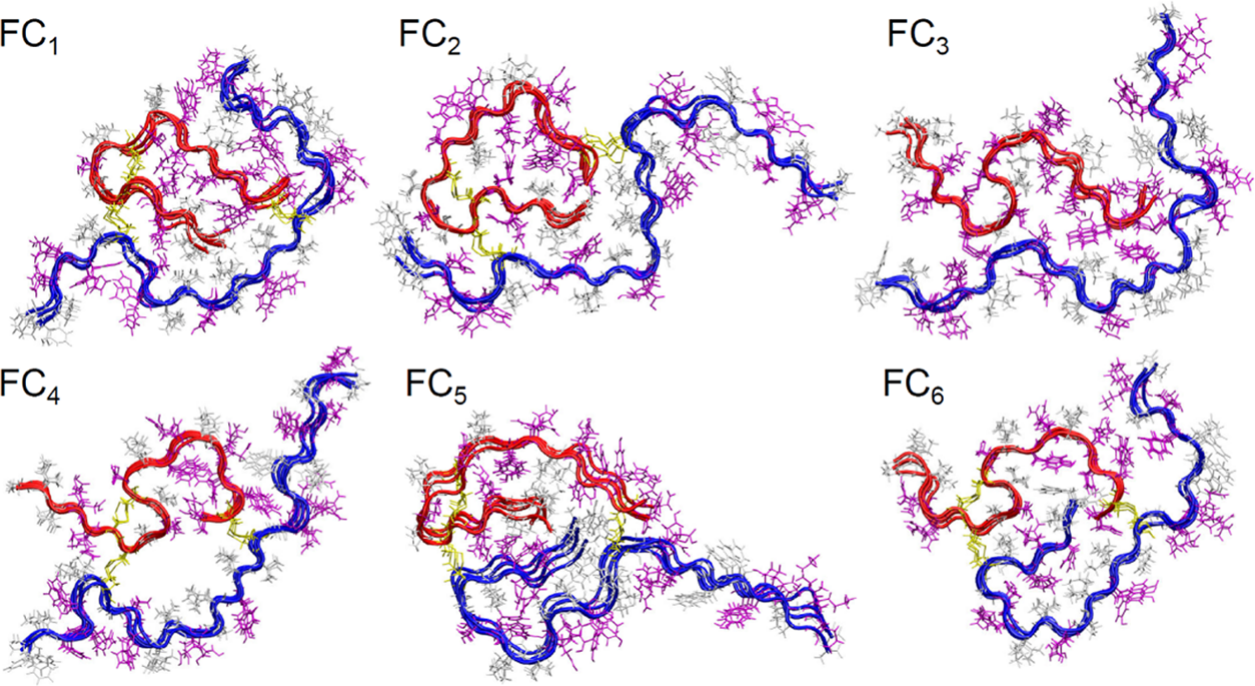
Initial models of insulin amyloidal assemblies obtained by in-register stacking of planarized conformers. Here, each model consists of four flat monomers. Hydrophobic side chains are colored in white; polar and charged residues are presented in magenta. The A- and B-chains are shown in red and blue, respectively; cysteine disulfide bridges are marked in yellow.

Before these flat insulin monomers were used as building blocks for amyloidal self-assembly, all conformers were optimized through 10 ns MD dynamics between two parallel surfaces in which only the repulsive part of the Lennard–Jones potential was employed. Selection of the best amyloid building blocks among the resulting unrestrained, yet maintaining planarity structures (in each topological category), was carried out according to the overall score of Ramachandran dihedral angles consistent with the extended structure (Methods). The docking of energetically optimized conformers was conducted by applying harmonic distance constraints between C_α_ atoms in aligned monomers. The process was repeated for the following layers, resulting in energy-optimized protofilament with two, three, and four layers. The assemblies of flattened conformers representing the six observed topological classes obtained after the initial conformational optimization using all-atom MD and explicit solvent model (Methods) are shown in Figure 3. The different packing regimes characteristic for the six stacked conformations limit the range of accessible inter- and intramolecular interactions, which is reflected in the contact maps calculated for these ensembles (see Figure S2 in the Supporting Information). For example, the stretching of the B-chain found in all, but FC_5_ and FC_6_ structures attenuate the intra-B-chain contacts, resulting in the absence of off-diagonal features in the corresponding range of the contact maps (Figure S2). The C-terminal segment of the A-chain forms close contact with the N-terminal sections of the A-chain (FC_1_, FC_2_, FC_5_) and B-chain (FC_5_) in the most densely packed structures. The interactions in the FC_3_ structure are dominated by interchain contacts, and it is the only case where the parallel alignment of both A- and B-chains involves their whole lengths. The FC_4_ structure contains a substantial void volume between separate midsections of A- and B-chains. Such a cavity, if preserved through the following stages of structural optimization, could cause either a significant van der Waals frustration or become filled with water molecules. On the other hand, the FC_5_ structure exhibits very dense packing with both N-terminal segments filling the central loop, although the proximity of two charged groups could prove to be a destabilizing factor.

**Figure 4:**
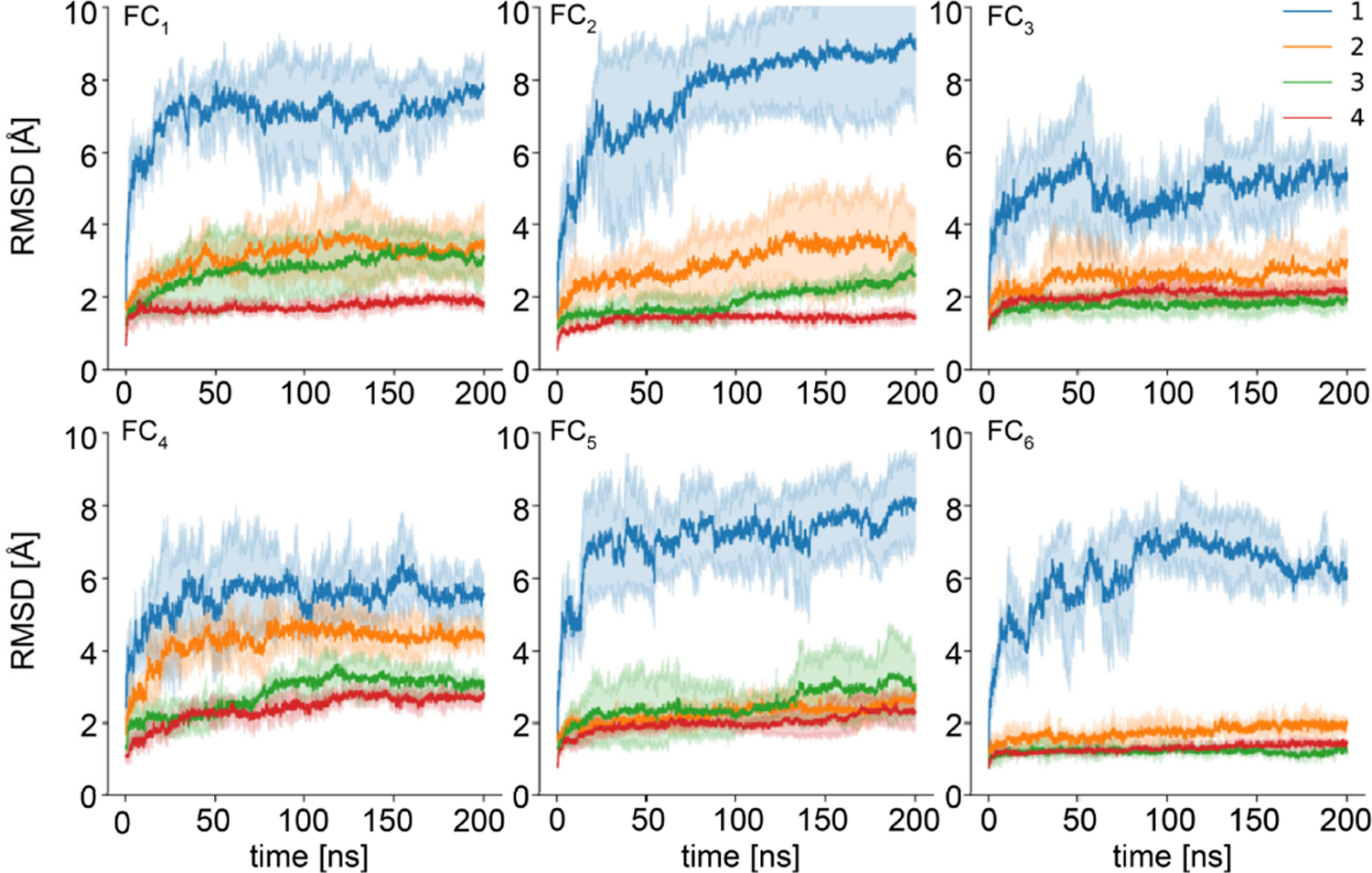
Stability of amyloidal stacks of the planarized insulin monomers in various 2D topologies probed by MD simulations at 300 K: temporal changes in the backbone C_α_ root-mean-squared deviation are impacted by the number of layers. Only C_α_ carbons between the interchain disulfide bonds are included in the calculation of RMSD (single layer, blue line; two layers, orange line; three layers, green line; four layers, red line). Thick lines represent the average values of the three independent runs. Topologies FC_3_, FC_5_, and FC_6_ exhibit similar constant stability for two, three, and four layers. A single layer of FC_3_ transiently adopts conformations similar to the starting structure.

Removal of the biasing restraints imposed on these assemblies at the early stages of simulations did not cause their dissociation and unfolding during prolonged MD simulations in an explicit solvent (Figure S3, Supporting Information). While the in-register alignment of parallel β-sheets was typically maintained for all topological classes, removal of the constraints triggered various changes in fibril’s morphology. Polymorphism of these assemblies arising from the distinct quasi-2D topologies of the ingredient insulin monomers has several consequential manifestations including the protofilament twist (Table 1), β-sheet content, presence of void volumes, and overall stability. The latter property is not only a function of the topological class of the building block but first and foremost of the number of stacked monomer layers. In Figure 4, the influence of the number of stacked insulin monomers belonging to the six quasi-2D-topological classes on the stability of assemblies is probed by measuring RMSD values of C_α_ carbons during isothermal all-atom MD simulations. For the sake of clarity, only C_α_ atoms confined to the disulfide-bond-restricted core amyloid region (residues Cys7A–Cys19A and Cys7B–Cys20B) were selected.

**Table 1:** Key Characteristics of Long Protofilaments Assembled from Planarized Insulin Monomers Belonging to Various Pseudo-2D Topological Categories^a^

category	inward directed termini	twist [deg.]	pitch [nm]	overall stability	H_2_O mol. per monomer
FC_1_	N- (A-chain)	0.1	1782	+	4.5
FC_2_	N-, C- (A-chain)	0.0		++	3.5
FC_3_	none	–0.9	198	++	3.5
FC_4_	C- (A-chain)	–1.1	162	+	>20
FC_5_	N- (A-chain), N- (B-chain)	ND	ND		2
FC_6_	C- (A-chain), N- (B-chain)	–0.1	1782	+++	1.5

a Due to the instability of FC_5_ fibrils, the twist and helical pitch could not be determined (ND). The approximate number of potentially sequestered (within the central topological loop) water molecules is given per insulin monomer.

In the absence of any constraints biasing the system to maintain the extended conformation, the single layers are entirely unstable: rapid increases in RMSD above 4 Å occur within the first 10 ns of the simulations irrespective of the topologic class. However, the single layer of FC_3_ transiently forms conformation similar to the one present in the fibril consisting of multiple layers. The stabilizing effect of layer multiplication is pronounced and universal, becoming very clear already for pairs of layers, especially for FC_2_, FC_5_, and FC_6_. Although the stretches of the B-chain outside the core region (residues Cys1B–Cys6B and Cys22B–Cys31B) are rather unstable, the core holds its initial state. With the addition of subsequent layers the stability builds up, however, the magnitude of these gains appear to be different for some structural variants (e.g., FC_3_, FC_5_, FC_6_, and FC_4_ fibrils are already quite stable as trimers and the effect of the fourth monomer is less pronounced). Importantly, the four-layered structures consistently maintain moderate RMSD values (<2 Å) throughout the simulation (in particular, FC_1_, FC_2_, and FC_6_). The presence of the FC_6_ structure among them is interesting given that only one planarization trajectory led to this topology.

**Figure 5:**
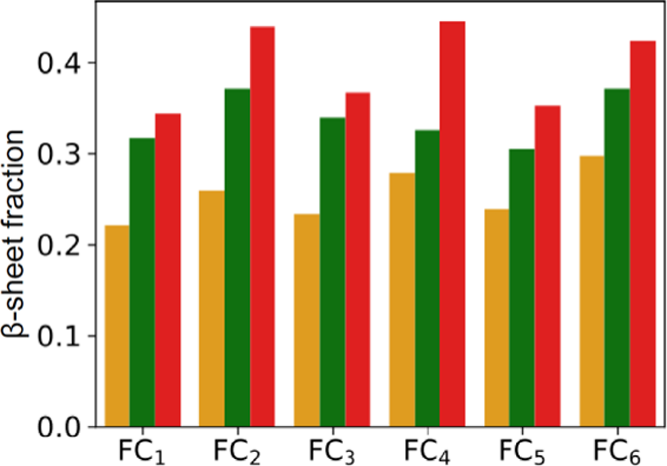
Average β-sheet content during the second part (100–200 ns) of simulations for various stacked quasi-2D topologies and different numbers of layers. The β-sheet content is defined as a ratio of the number of the C_α_ carbons involved in the β-sheet structure (as recognized by the Stride algorithm) to all of the C_α_ carbons in the molecule (two layers, orange bars; three layers, green bars; four layers, red bars).

Steadily low RMSD values point to an elevated structural order, which would be consistent with high β-sheet content. The calculated ratios of backbone atoms involved in the canonical β-sheet conformation (as defined by the Stride algorithm^44^) during the final 100 ns of the MD simulations are reported in Figure 5.

**Figure 6:**
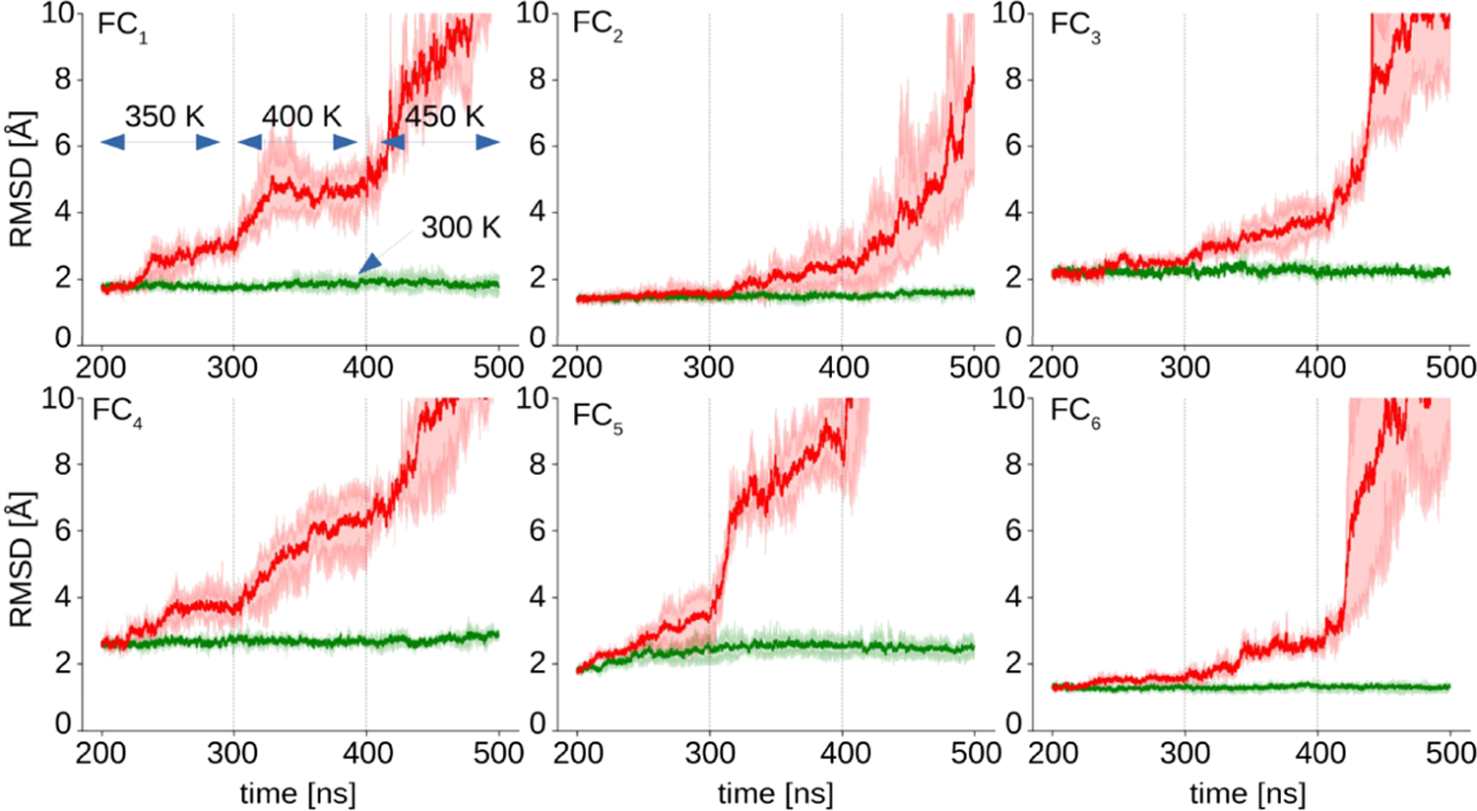
Relative stability of quadruple layer stacks of various topologies probed by changes in RMSD during high-temperature unfolding. These simulations correspond to prolonged MD runs of the simulations shown in Figure 4. Only C_α_ carbons between the interchain disulfide bonds are included in the calculation of RMSD. The temperature was maintained at 350 K for the initial 100 ns followed by jumps to 400 K and finally 450 K, as indicated with vertical dashed lines. Thick red lines denote average RMSD values from the three independent simulations. Green lines represent the average RMSD values from the control runs at 300 K.

Unsurprisingly, in all of these cases, β-sheet becomes more abundant with the increasing number of stacked monomers. The three top-scoring variants are FC_2_, FC_4_, and FC_6_: along with the two more stable structures, the relatively fluctuation-prone FC_4_ type reveals a significant presence of β-sheet. We have conducted an additional survey of stability of four-layered amyloidal assemblies of all six topological variants by subjecting them to temperature ramp MD simulations summarized in Figure 6.

The simulations were carried out at 350 K for the first 100 ns followed by the temperature jumps to 400 and 450 K (at 400 ns), while control simulations were run at 300 K. Based on the behavior at 350 K, the six amyloid variants can be placed in the following approximate order of decreasing stability: FC_2_, FC_6_ > FC_3_, FC_5_ > FC_4_, FC_1_. Furthermore, assemblies of FC_2_, FC_3_, and FC_6_ appear to be rather resistant to temperature increases to 400 K contrasting with the fast structural disruption caused in the other three isomers. Unfolding at 450 K is the fastest for the FC_5_ aggregate. The data presented so far suggests that the FC_2_ and FC_6_ assemblies are particularly stable and that this stability coincides with the increased β-sheet content. What distinguishes both these isomers on the structural level is the inward placement of N- and C-termini (belonging to a single A-chain in the case of FC_2_ and B- and A-chains in FC_6_) in the core region (Figures 2C and 3). Hypothetically, the proximity of N- and C-termini could lead to a salt-bridge-like interaction between them, which could contribute strongly to the overall stability of the aggregate.^48,49^

**Figure 7:**
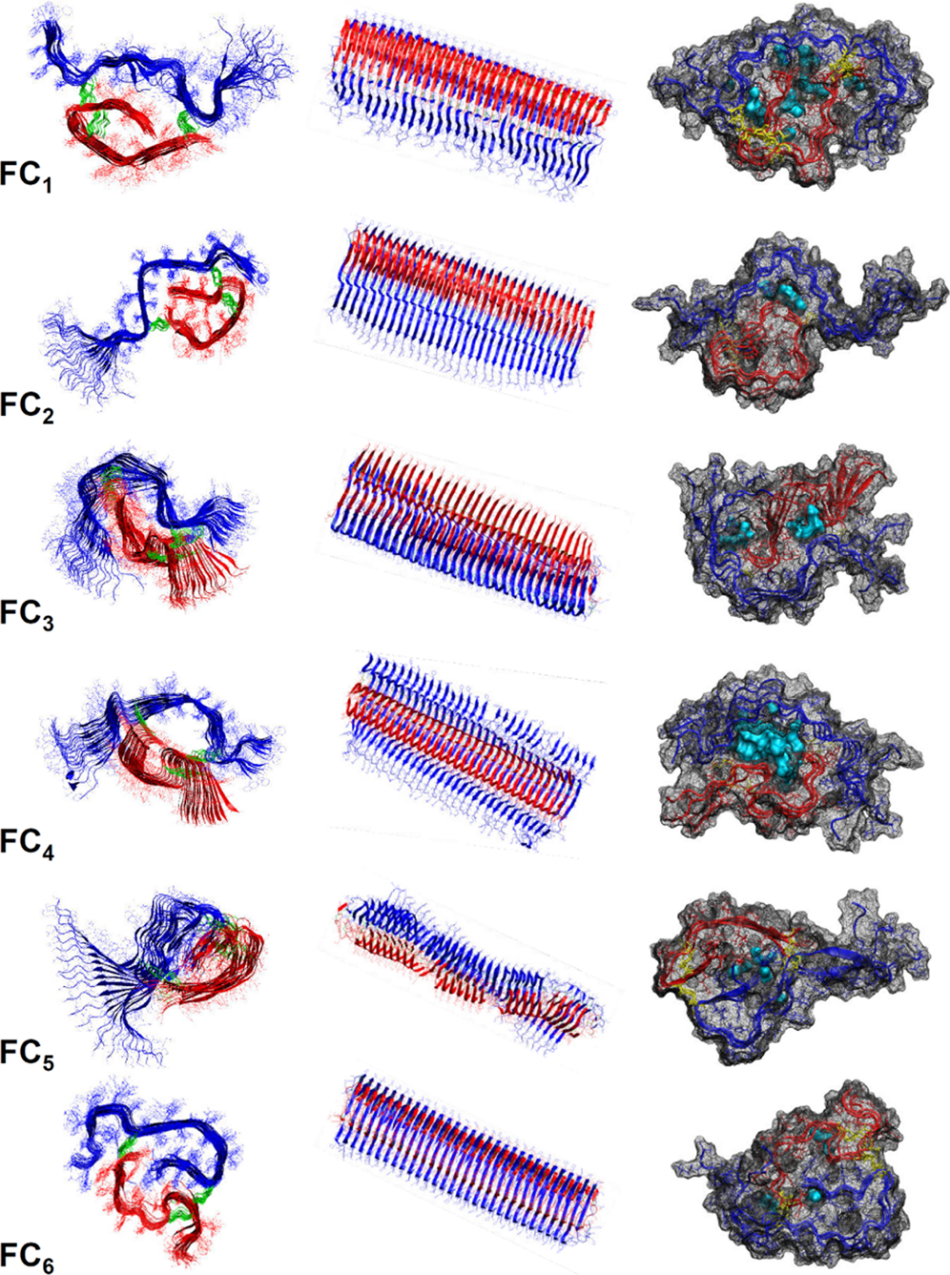
Insulin amyloid structures obtained by in-register stacking (followed by molecular dynamics in implicit solvent) of 30 planarized monomers of indicated quasi-2D topologies represented by final models after 100 ns simulations. A- and B-chains are marked in red and blue, respectively. In the right column, the final structures from the explicit solvent MD simulations are shown: the internal cavities within fibrils are filled with water molecules (light blue). The amounts of the sequestered water in the internal canals of FC_1_...FC_6_ fibrils vary significantly (Table 1).

To assess the structural features of protofilaments consisting of different insulin monomers obtained in this study, we also built longer filaments composed of 30 monomer layers. Cross sections and side views of such protofilament structures obtained for the six different quasi-2D topologies are presented in Figure 7 along with the visualizations of internal cavities that could be filled with water molecules. In general, these multilayer structures proved to be remarkably stable during the simulations with the exception of FC_5_ where the N-terminal fragments of both chains are repulsed from each other causing significant disruption. There are marked differences in terms of fibril’s twist and the number of water molecules that could be possibly sequestered within the central topological loop (Figure 7 and Table 1). The FC_3_ and FC_4_ fibrils reveal a negative (left-handed) twist of approximately ∼1° per monomer layer. For other structures, the obtained twist values are either even lower (FC_1_, FC_2_, FC_6_) or impossible to determine due to the fibril’s instability (FC_5_). These values translate into very long helical pitches (Table 1), which contrast with those observed experimentally for mature insulin amyloid fibrils (∼240 nm^32^). However, we have shown earlier that the thinnest fibrillar specimen of insulin amyloid (which correspond most likely to individual protofilaments, as simulated in this study) may be essentially flat, according to atomic force microscopy (AFM),^32^ while the superstructural twist (which is most accessible to AFM) of several intertwined protofilaments is an outcome of the complex interplay of various factors including a twist of component protofilaments.^50^

In the case of FC_1_, FC_2_, and FC_6_ fibrils, the negligible twist correlates with either A- or B-chain’s N-termini being placed inside of the central loop. Hypothetically, fully extended (as in FC_3_ and FC_4_ fibrils) A- and B-chains could adopt more easily the conformational twist that is intrinsic to the canonical parallel β-sheet structure. However, should either chain become bent with its N-terminal fragment directed inward to the loop’s center (FC_1_, FC_2_, FC_5_, FC_6_), the conflicting torque forces acting on the sections of A- and B-chains could diminish the effective twist of the whole protofilament. Additional studies are needed to assess the plausibility of this scenario and its involvement in nontwisted insulin fibrils.^32,51^

All of the mature structures examined in this study contained core void volumes that could be filled with at least a few water molecules (per monomer, Figure 7, right column, and Table 1). There have been conflicting reports on the presence of water within amyloid fibrils. Some studies postulated that amyloid fibrils are densely packed interfaces that are generally dry,^15^ while others suggested that the amyloid core is loosely packed^52,53^ and therefore permissive to being penetrated by and filled with water.^54,55^ Our previous experimental studies suggested that bovine insulin amyloid fibrils grown under typical conditions are devoid of sequestered water.^56,57^ By applying the criterion of hydration of the internal canal, the six amyloid structures examined here could be divided into three groups: (i) dry fibrils (FC_5_ and FC_6_) containing 1–2 sequestered water molecules per monomer, (ii) moderately wet fibrils (3–5 water molecules per monomer in FC_1_, FC_2_, FC_3_), and (iii) water-soaked amyloid (more than 20 H_2_O molecules per insulin monomer in the case of FC_4_ fibrils), which is clearly least consistent with the aforementioned experimental studies.

**Figure 8:**
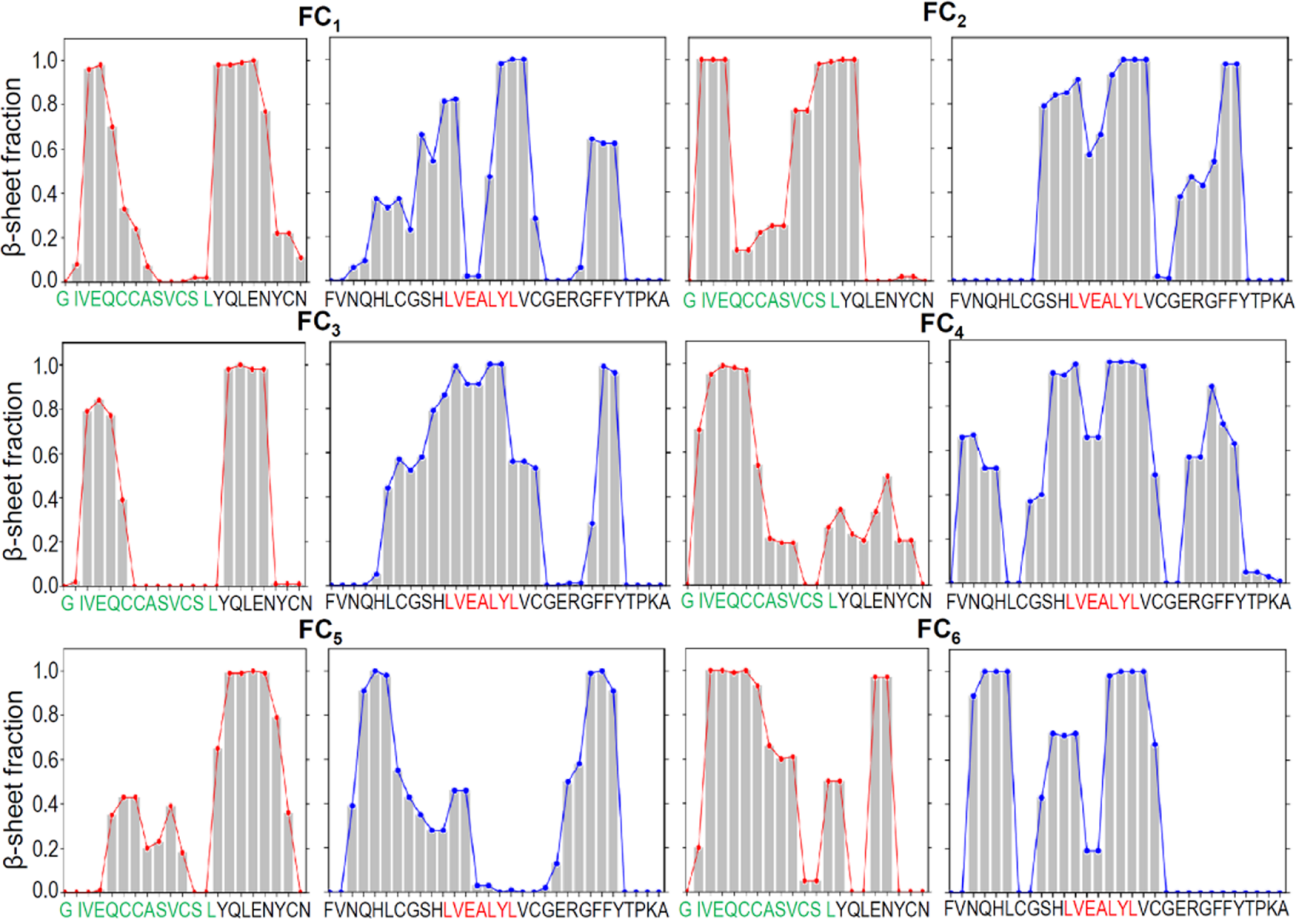
Local β-sheet content for amyloidal quadruple insulin monomer layers in various quasi-2D topologies along the amino acid sequence (β-sheet content is defined as a fraction of MD simulation time in which particular residue is assigned to this conformation). The calculations were based on averaging the local conformational state over three independent MD runs (final 100 ns of a 200 ns long simulation). The LVEALYL segment implicated in the insulin amyloid steric zipper is red-marked, whereas the strongly amyloidogenic N-terminal fragment of the A-chain is marked in green.

Previous studies pointed to the importance of certain regions of the insulin’s covalent structure for the transition to amyloid fibrils. The LVEALYL section of the B-chain is likely to be involved in the formation of a steric zipper stabilizing the fibrils,^58^ whereas the disulfide-bond N-terminal fragment of the A-chain encompassing the first 11–13 amino acid residues has a very strong propensity to form amyloid.^59−63^ In the fibrillar state, both these segments are expected to form stable β-sheet conformations. From this perspective, it becomes crucial to look at the stability and β-sheet content of the FC_1_...FC_6_ four-layer assemblies at the resolution of individual amino acid residues. Figure 8 provides an insight into the local propensity to form a β-sheet structure along the amino acid sequence of insulin with respect to the four-layer insulin monomer assemblies. We note that while there is not a single structure saturated in the β-sheet content at both these segments simultaneously, some topologies (FC_2_, FC_4_, FC_6_) perform better than others (FC_1_, FC_3_, FC_5_). In Figure S4 (Supporting Information), the parallel data on molecular fluctuations is presented.

**Figure 9:**
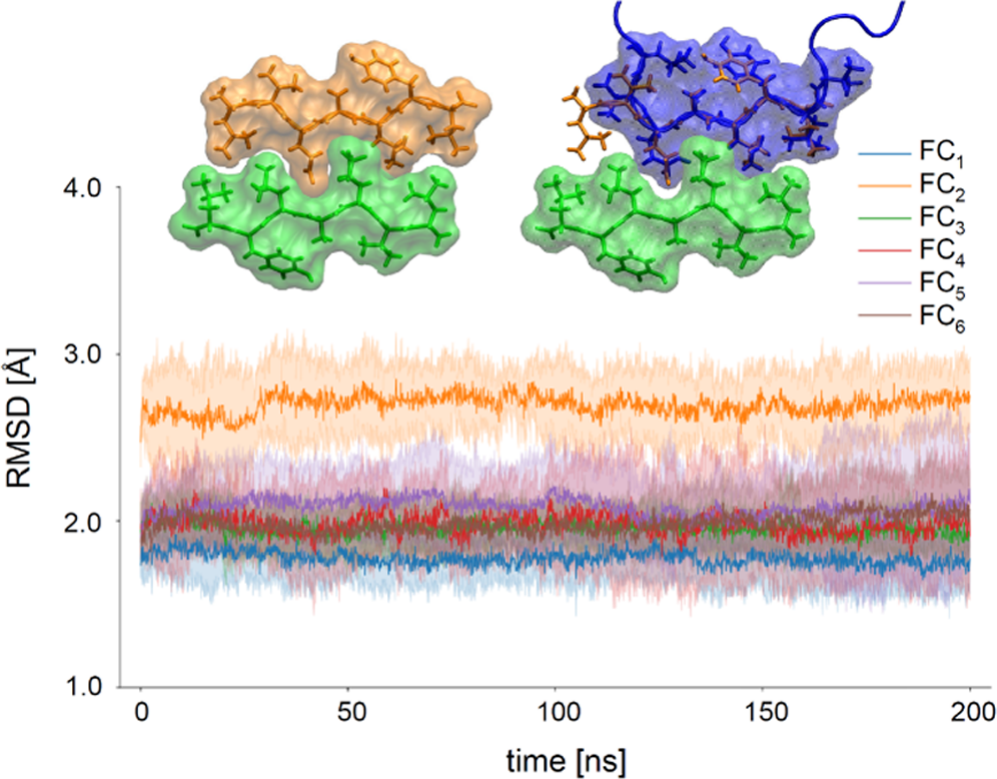
Similarity between LVEALYL segments: as a part of the insulin’s steric zipper (PDB: 3HYD) and a part of FC_1_...FC_6_ fibrils (C_α_ RMSD profiles for all topologies). Thick lines represent the average of the three independent, 200 ns simulations. Inset: in the left panel, the crystal structure of LVEALYL fibrils (3HYD) is presented; the orange sticks and orange surface represent one β-strand, whereas the green surface and green sticks depict the other β-strand jointly forming the steric zipper; in the right panel, an example of the structural alignment between the 3HYD peptide and the LVEALYL segment in the FC_3_ topology is shown.

We also looked at structural similarities between LVEALYL segments: as a part of the steric zipper (PDB: 3HYD,^58^) and as part of simulated fibrillar structure (Figure 9). The requirement to form the steric zipper appears to be best satisfied by the topologies of FC_1_ (C_α_ RMSD < 2.0 Å), FC_3_ and FC_4_, followed by FC_6_, FC_5_, and finally FC_2_ in which the turn around the residue Glu3B causes the main dissimilarity (C_α_ RMSD ∼ 3.2 Å). Interestingly, the structures become nearly identical (C_α_ RMSD ∼ 0.5 Å) when the leucine residue of the LVEALYL segment is neglected. Overall, the assemblies most consistent with the expected β-sheet presence in the N-terminal region of the A-chain are those of FC_6_ and FC_4_ followed by FC_2_, FC_1_, FC_3_, and FC_5_ (although molecular fluctuations in these segments are strongly dumped in FC_2_ and FC_1_).

## 4 Conclusions

In conclusion, here, we have put forward and examined an alternative approach to the exceedingly challenging problem of *in silico* simulations of amyloid structures formed by proteins with complex topologies. In our approach, monomers of insulin, selected as a model amyloidogenic protein with a topology constrained by disulfide bonds, were first “planarized” and subsequently used as building blocks for amyloid aggregates. Through this protocol, six different amyloid structures varying in pseudo-2D topologies of flat insulin monomers were accessed. These structural variants were analyzed in terms of overall stability and compatibility with the existing low-resolution experimental data on insulin amyloid fibrils as well as high-resolution structural data on fibrils derived from short insulin fragments. One of the here-presented plausible structural variants of insulin fibrils, marked as FC_6_, is distinct in terms of the N-terminal part of insulin’s B-chain being pointed inward to the center of the main topological loop of flattened insulin monomer with B-chain’s C-terminus and A-chain’s both termini protruding outside the fibril. The FC_6_ variant is remarkably stable and presents a significant β-sheet content, especially in the regions expected to acquire this fold upon fibrillization, namely, the steric zipper LVEALYL region, and the N-terminal fragment of the A-chain. The variant is also largely devoid of internally trapped water molecules, which is also in accordance with the earlier experimental studies. Remarkably, the building block topology for the FC_6_ variant is the rarest among the all planarized insulin conformers. Different stages of our multiscale approach may be easily modified and supplemented by various computational methods—for example, by coupling to molecular docking tools such as CABS-dock.^64^ As the phenomenon of amyloidogenesis is not restricted to simple single-chain proteins and peptides, further efforts are urgently needed to create tools to develop viable molecular structures of amyloid fibrils formed by proteins with complex topologies.

## Supporting Information Available

PDB files of FC_1_...FC_6_ amyloid structures consisting of ten insulin monomer layers (ZIP)
Additional data on the structure and stability of FC_1_...FC_6_ amyloid fibrils and the impact of the initial spatial orientation of the insulin monomer’s on the outcome of planarization (PDF)
