# Supplementary material for: Virtual Quasi-2D Intermediates as Building Blocks for Plausible Structural Models of Amyloid Fibrils from Proteins with Complex Topologies: A Case Study of Insulin
Source: Langmuir. 2022 May 26;38(22):7024–34. doi: 10.1021/acs.langmuir.2c00699 (PMC9178918; doi:10.1021/acs.langmuir.2c00699)
Supplement: Supplementary file 2 — la2c00699_si_002.pdf [file la2c00699_si_002.pdf]

Supporting Information

for

Virtual quasi-2D intermediates as building blocks for plausible structural models of amyloid fibrils from proteins with complex topologies: A case study of insulin

by

Wojciech Puławski[1,2*] and Wojciech Dzwolak[1,3*]

[1]*Institute of High Pressure Physics, Polish Academy of Sciences, Sokołowska 29/37 Str., 01-142 Warsaw, Poland*
[2]*Bioinformatics Laboratory, Mossakowski Medical Research Institute, Polish Academy of Sciences, 5 Pawinskiego Street, 02-106 Warsaw, Poland (Present address)*
[3]*Faculty of Chemistry, Biological and Chemical Research Centre, University of Warsaw, 1 Pasteur Str., 02-093 Warsaw, Poland.*

[*] Corresponding authors:
W. Puławski: Phone: (+48) 22 849 93 58, E-mail: wpulawski@imdik.pan.pl
W. Dzwolak: Phone: (+48) 22 552 6567, E-mail: wdzwolak@chem.uw.edu.pl

Contents

A. Impact of the initial spatial orientation of the insulin monomers on the outcome of the *in silico* planarization ……………………………..……………... S2
B. Contact maps for flattened insulin monomers of various topological classes………………………………………………………….……….…….……S3
C. Long simulations of four layer aggregates under the ambient conditions....... S4
D. Fluctuations of C_α atoms of insulin residues within four layer aggregates…...S5

A. <u>Impact of the initial spatial orientation of the insulin monomers on the outcome of the *in silico* planarization.</u>

The starting conformation of the folded insulin monomer was extracted from the hexamer structure of bovine insulin (PDB, entry 2A3G). Prior to the planarization MD procedure described in Methods six distinct starting spatial orientations of the monomer were obtained by its stepwise 90-degree rotations around X, Y, and Z axes. One hundred independent planarization simulations were carried out for each initial spatial orientation of the insulin monomer.

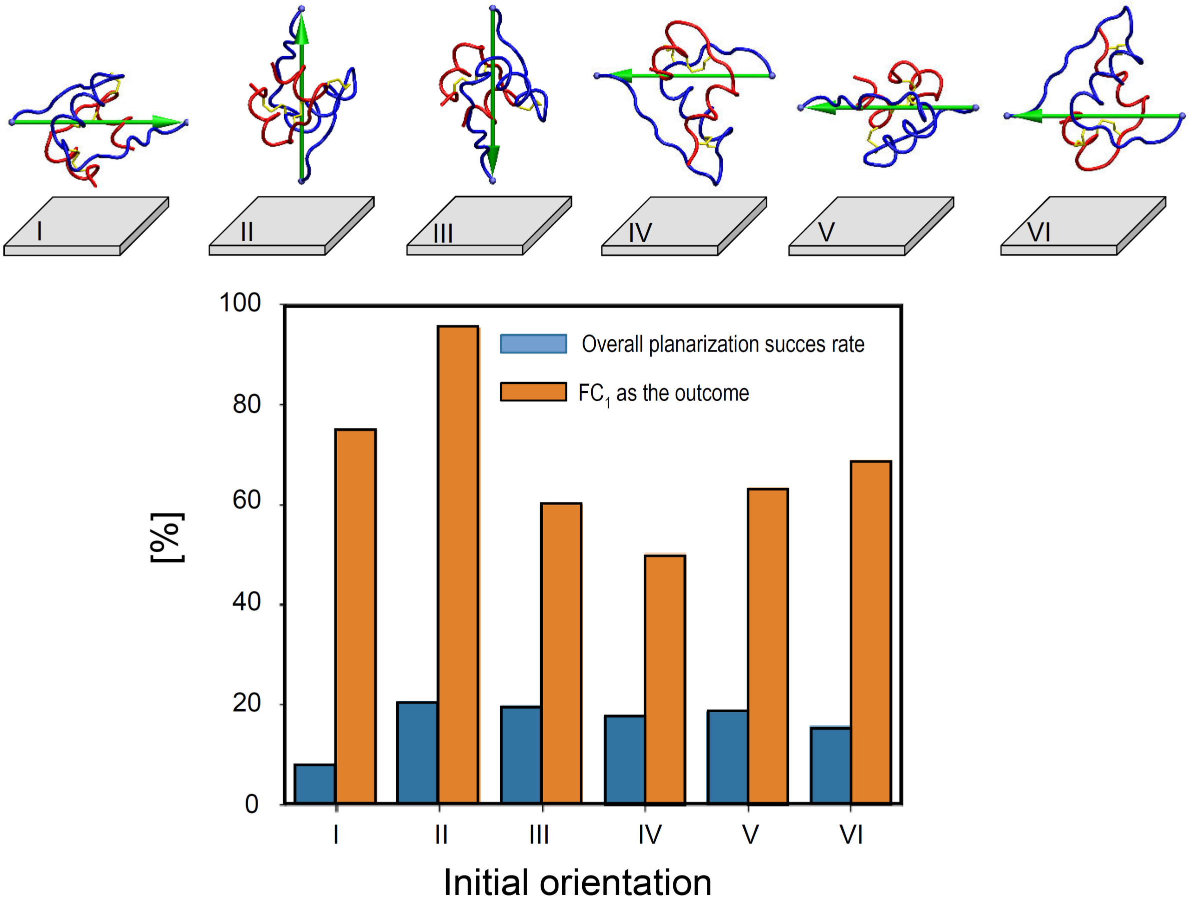

Figure S1. *Impact of the initial orientation on the success rate and topological outcome of the planarization. Upper panel: a simplistic representation of the six various initial orientations (I..VI) of the natively folded insulin monomer (PDB entry 2A3G) vs. the surface on which the monomer was planarized. The green arrow represents an arbitrarily selected molecular axis connecting N- and C-ends of the B-chain. Bottom panel: the dark blue bars indicate the percentage of successful (i.e. leading to bump-free flat conformers of any topological class) simulations for the indicated initial orientation of the monomer; the orange bars represent ratio (per cent) of successfully planarized conformers that were classified as the most common FC_1 pseudo-2D-toplogy.*

The data shown in **Fig. S1** indicates that [**i**] the planarization often leads to imperfectly flattened conformers (i.e. containing specific for this type of simulation kinetic traps: 'bumps' of persistently superimposing main chains and / or disulfide bonds), and [**ii**] that the overall planarization success does not exceed 20 % (less than 10 % for orientation I). Regardless of the initial orientation, FC_1 was the most common pseudo-2D topology among the successfully planarized monomers. The relative frequency of FC_1 appears to depend on the initial spatial orientation (e.g. is nearly twice more likely for orientation II than IV).

B. Contact maps for flattened insulin monomers of various topological classes.

The contact maps were calculated for single flattened conformers representing the six topological classes after the initial conformational optimization using all-atom MD and explicit solvent model (Methods) shown in Fig. 3 of the main article. The different packing regimes characteristic for the stacked conformations limit the range of accessible inter- and intramolecular interactions The extension of the B-chain found in all pseudo-2D topologies except for FC_5 and FC_6 attenuates the intra-B-chain contacts leading to the disappearance of the off-diagonal features in the corresponding range of the contact maps (**Fig. S2**). The C-terminal segment of A-chain forms close contacts with the N-terminal sections of A-chain (FC_1, FC_2, FC_5) and B-chain (FC_5) in the most densely packed structures. The interactions within the FC_3 structure are dominated by interchain contacts (this is the only case where the parallel alignment of both A and B chains involves their whole lengths of these chains). The FC_4 structure contains a substantial void volume between separate midsections of A- and B-chains. Such a cavity, if preserved through the following stages of structural optimization, could cause either a significant van der Waals frustration, or be filled with water molecules. On the other hand the FC_5 structure exhibits very dense packing with both N terminal segments filling the central loop, although the proximity of two charged groups could prove to be a destabilizing factor.

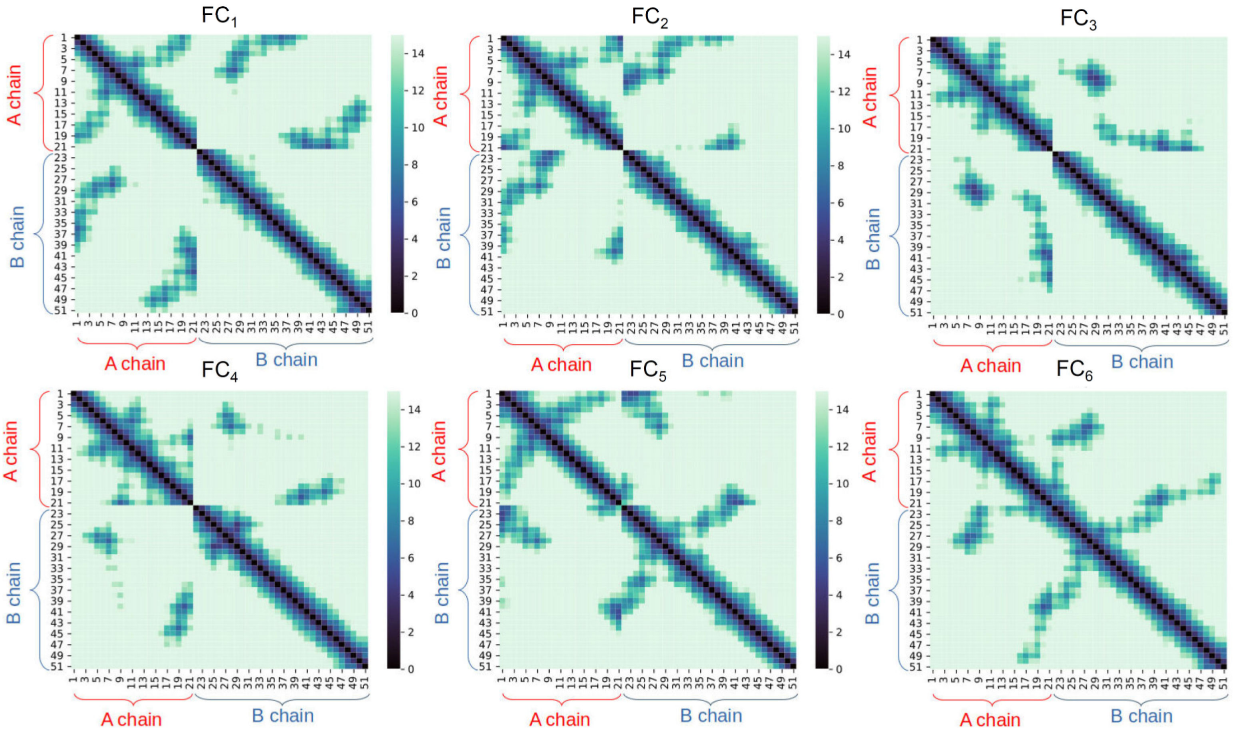

Figure S2. Contact maps (distance in [Å] between C_α atoms of various residues) calculated for the flattened conformers representing the six observed topological classes, as indicated. The numbers along the axes correspond to odd residues of insulin's A-chain (1..21) and B-chain (23..51).

C. Long simulations of four layer aggregates under the ambient conditions.

The stability of four layer aggregates representing the six pseudo-2D topological classes has been probed through 2 μs-long all atom MD simulations in explicit solvent at 300 K (see Methods). In **Fig. S3**, time-dependent changes in RMSD (C_α atoms) and β-sheet content (as percentage of residues involved in the β-fold) are plotted. The structures adjacent to each data panel are snapshots taken at the end of each simulation. FC_2, FC_3 and FC_6 structures are particularly stable and retain the high β-sheet contents throughout the simulations. The lowered β-sheet content observed for FC_1 and FC_5 correlates with the structural instability reflected by the RMSD drifting to higher values.

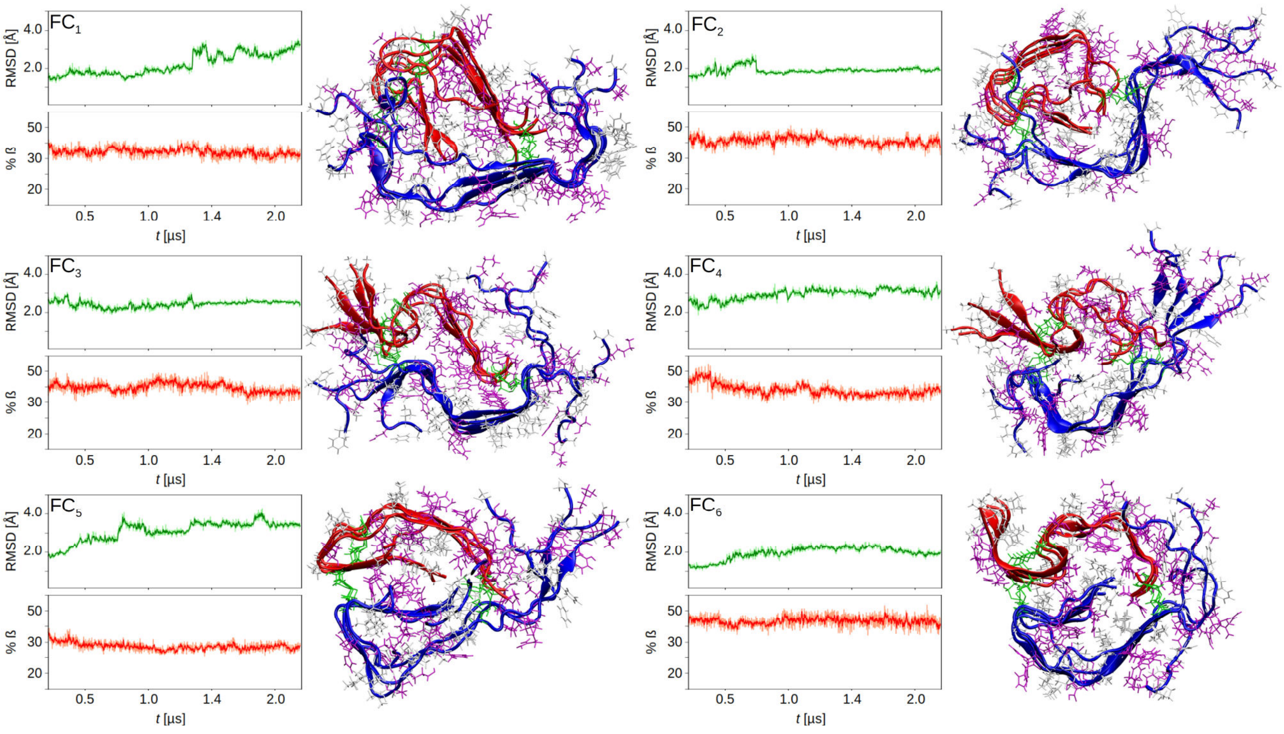

Figure S3. Long MD simulations and the resulting structures of the various four layer aggregates of planarized insulin monomers in various pseudo-2D topologies: C_α RMSD (green) and β-sheet fraction (red). Only C_α atoms of the main chain segments constituting the central loop (formed by middle sections of both chains and Cys7A-Cys7B / Cys19B-Cys20A disulfide bonds) are taken into account. The β-sheet content is defined as percentage of C_α carbons involved in the β-structure, as recognized by the Stride algorithm.

D. Fluctuations of C_α atoms of insulin residues within four layer aggregates.

Root mean square fluctuations (RMSF) for C_α atom at various residues were calculated by averaging of the local conformational state over three independent MD runs (second half (100 ns) of the 200 ns simulations) to assess the rigidity of four layer aggregates (**Fig. S4**). The data parallels that shown in Fig. 8. The inward placement of N- and C-terminal main chain segments significantly dampens the molecular fluctuations, as is the case of, for example, A-chain's N-terminus in FC_1 compared to FC_3. The middle sections of main chains reveal uniformly attenuated levels of fluctuations. This is particularly clear for the steric-zipper-forming LVEALYL section of B-chain (e.g. FC_6). We note low RMSF values for the amyloidogenic N-terminal part of A-chain (GIVEQCCASVCSL) in the case of FC_1 and FC_2.

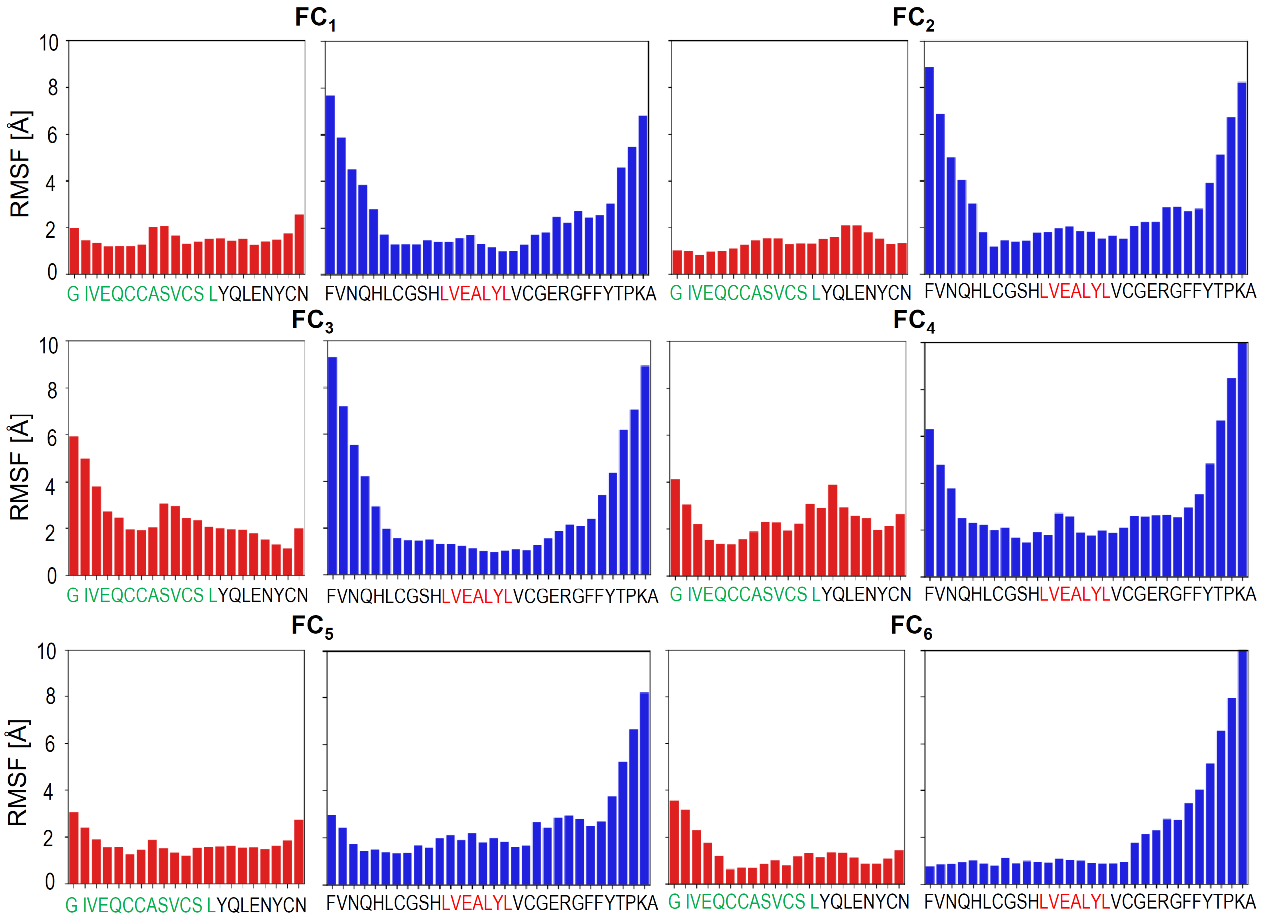

Figure S4. Root mean square fluctuations obtained for each residue's C_α atom. Calculations were carried out for four layer insulin aggregates belonging to different pseudo-2D topological categories and were based on averaging of the local conformational state over three independent MD runs (second half of 200 ns long isothermal simulation). The LVEALYL segment implicated in insulin amyloid steric zipper is red-marked whereas the strongly amyloidogenic N-terminal fragment of A-chain is marked in green. The data supplements Fig. 8
